# Severity of injuries in different modes of transport, expressed with disability-adjusted life years (DALYs)

**DOI:** 10.1186/1471-2458-14-765

**Published:** 2014-07-29

**Authors:** Marko Tainio, Dorota Olkowicz, Grzegorz Teresiński, Audrey de Nazelle, Mark J Nieuwenhuijsen

**Affiliations:** Systems Research Institute, Polish Academy of Sciences, Ul. Newelska 6, Warsaw, 01-447 Poland; UKCRC Centre for Diet and Activity Research (CEDAR), MRC Epidemiology Unit, University of Cambridge, Cambridge, UK; The Faculty of Power and Aeronautical Engineering, Warsaw University of Technology, ul. Nowowiejska 24, Warsaw, 00-665 Poland; Department of Forensic Medicine, Medical University of Lublin, ul. Jaczewskiego 8, Lublin, 20-090 Poland; Centre for Environmental Policy, Imperial College London, 13 G7 Princes Gardens, London, SW7 1NA UK; Center for Research in Environmental Epidemiology (CREAL), Parc de Recerca Biomèdica de Barcelona – PRBB, C. Doctor Aiguader, 88, Barcelona, 08003 Spain

**Keywords:** Traffic, Transport, Fatality, Crash, Injury, DALY, YLD, YLL, AIS, HIA

## Abstract

**Background:**

Health impact assessment (HIA) studies are increasingly predicting the health effects of mode shifts in traffic. The challenge for such studies is to combine the health effects, caused by injuries, with the disease driven health effects, and to express the change in the health with a common health indicator. Disability-adjusted life year (DALY) combines years lived disabled or injured (YLD) and years of life lost (YLL) providing practical indicator to combine injuries with diseases. In this study, we estimate the average YLDs for one person injured in a transport crash to allow easy to use methods to predict health effects of transport injuries.

**Methods:**

We calculated YLDs and YLLs for transport fatalities and injuries based on the data from the Swedish Traffic Accident Data Acquisition (STRADA). In STRADA, all the fatalities and most of the injuries in Sweden for 2007–2011 were recorded. The type of injury was recorded with the Abbreviated Injury Scale (AIS) codes. In this study these AIS codes were aggregated to injury types, and YLDs were calculated for each victim by multiplying the type of injury with the disability weight and the average duration of that injury. YLLs were calculated by multiplying the age of the victim with life expectancy of that age and gender. YLDs and YLLs were estimated separately for different gender, mode of transport and location of the crash.

**Results:**

The average YLDs for injured person was 14.7 for lifelong injuries and 0.012 for temporal injuries. The average YLDs per injured person for lifelong injuries for pedestrians, cyclists and car occupants were 9.4, 12.8 and 18.4, YLDs, respectively. Lifelong injuries sustained in rural areas were on average 31% more serious than injuries in urban areas.

**Conclusions:**

The results show that shifting modes of transport will not only change the likelihood of injuries but also the severity of injuries sustained, if injured. The results of this study can be used to predict DALY changes in HIA studies that take into account mode shifts between different transport modes, and in other studies predicting the health effects of traffic injuries.

**Electronic supplementary material:**

The online version of this article (doi:10.1186/1471-2458-14-765) contains supplementary material, which is available to authorized users.

## Background

Health Impact Assessment (HIA) studies, and other similar assessment studies, are increasingly combining health effects of different stressors. HIA studies assessing the health effect of transportation often consider, and combine, health effects caused by traffic crashes, air pollution, noise, physical activity and many other stressors [1–5].

The challenge for HIAs is to combine positive and negative health effects, and to express these effects with one or more indicators of health. From the data and computational point of view the easiest indicator is premature mortality, with different variations (applied e.g. by the studies of de Hartog et al. [3] and Rojas et al. [1]). The problem with a premature mortality indicator is that it does not take into account the victim’s age. This might cause misleading impressions of health burden if different stressors are affecting population with different ages. The other problem with a mortality indicator is that for some stressors, such as fine particulate matter (PM_2.5_) air pollution, mortality captures most of the combined health effects of mortality and morbidity, whereas for some other stressors, such as lead, the morbidity effect is as important as, or larger, than the mortality impact [6, 7].

One increasingly popular indicator of health is the disability-adjusted life-years (DALY) measure. The DALY method was developed for the Global Burden of Disease studies [8–10] and it has been used in a number of transport related HIA studies (e.g. [1, 2, 5, 11]). The DALY is a health-gab measure that compares the current health to an ideal situation where everyone lives a long life without any diseases or disabilities. The calculation of DALYs has two components: years of life lost due to premature mortality or fatality (YLLs) and years lived disabled or injured (YLDs). The YLLs are calculated by comparing the age of the deceased person to predicted life-expectancy of a person with same age and gender. YLDs are calculated by multiplying the number of diseases with the disability weight and the duration of that disease.

The calculation of YLDs for transport injuries requires detailed information on the injury types, disability weight and the duration of injuries caused by crashes. All these three components vary in time and space, and it is also likely that these components vary between modes of transport. That means that the severity of injuries, when expressed with YLDs, could be different for those traveling in a car and cyclists, and this difference might be important when estimating the health burden of mode shifts. However, transport mode specific YLDs are rare. The Dhondt et al. [12] study from Belgium is the only one, that we are aware of, that has estimated YLDs per person for different modes of transport and in that study only four victim groups (drivers, passengers, bus occupants and slow mode victims) were analysed, limiting the use of the results in the analyses with several transport modes.

In this study we calculate the average YLDs that one injured person suffers in a traffic crash. The results are calculated separately for different modes of transport, gender and age. The location of the crash is also taken into account by comparing the average YLDs in urban and rural areas. The main focus is on injuries sustained by pedestrians, cyclists and car users (both drivers and occupants), but other modes of transport are also calculated for comparison. For comparison, we also calculate YLLs from fatalities for the same population and years to compare the importance of injuries with fatalities. This study does not investigate the underlying reasons for the severity differences. The main purpose is to calculate YLDs and represent the results in such a way that future HIA studies of transport scenarios could benefit from the results.

## Methods

The average DALYs per person is calculated based on transport injury data from Sweden. The Swedish injury data was selected because it has detailed information on the transport modes of victims, injuries sustained by victims and on the location of the crash. The DALY indicator was selected because of its increasing popularity as a health indicator and because the results could be compared with the similar injury burden studies [12–17]. The data, assumptions and calculations are described below.

### Injury data

The Global Burden of Disease studies from 1996 [8] and 2006 [18] grouped injuries caused by traffic crashes to 33 short-term and lifelong injuries, and then calculated YLDs individually for each injury. To estimate the average YLDs for different modes of transport, we first predicted the injury type variation between different kinds of crashes. For this we used the Swedish Traffic Accident Data Acquisition (STRADA) database. STRADA is a national database that records both transport injuries and crashes in Sweden, and it combines data from both police and hospital records [19]. Police records include all road crashes with injuries, and hospital records include all emergency room visits from hospitals reporting to STRADA. Approximately 64% to 89% of hospitals in Sweden were reporting to STRADA in the years 2007 and 2011, respectively (Table 1). The injuries were recorded with International Classification of Diseases (ICD) diagnoses and Abbreviated Injury Scale (AIS)-codes. STRADA data has been used in a number of transport injury studies [20, 21].

**Table 1:** **Number of injuries and fatalities for different gender, age, year, location and transport modes**

	Injury data (number of victims)	%	Fatality data (number of victims)	%
All	159,352	100%	1811	100%
Gender (i)				
Male	79,102	50%	1336	74%
Female	80,250	50%	475	26%
Age (in years) (j)				
0-20	38,310	24%	281	16%
21-40	45,114	28%	509	28%
41-60	41,208	26%	469	26%
61-80	28,235	18%	394	22%
81-100	6,457	4%	158	9%
>100	28	0%	0	0%
Year (m)				
2007	26,155	16%	471	26%
2008	28,781	18%	397	22%
2009	30,222	19%	358	20%
2010	36,300	23%	266	15%
2011	37,894	24%	319	18%
Location of the crash (l)				
Urban	100,663	63%	462	26%
Rural	41,444	26%	1299	72%
Unknown	17,245	11%	50	3%
Mode of the transport (k)				
Pedestrian	47,261	30%	231	13%
Bicycle	36,973	23%	125	7%
Other active travel mode	2,218	1%	0	0%
Moped	9,735	6%	55	3%
Motorcycle	5,489	3%	241	13%
Car	52,728	33%	1038	57%
Truck	1,363	1%	71	4%
Bus	1,691	1%	11	1%
Other, unknown, missing	1894	1%	39	2%
Coverage of STRADA data (%)				
2007	64%		100%	
2008	70%		100%	
2009	74%		100%	
2010	82%		100%	
2011	89%		100%	

Description of different transport modes is in Table S2 (Additional file 1).

From STRADA we obtained all the recorded transport injuries for 2007–2011 (Table 1). During these years, 159 352 persons sustained 258 572 individual injuries. The number of injuries per injured person varied between 1 and 26. For each injury, the anatomical location and severity were described with the AIS codes (year 2005 version). The AIS is an anatomic based coding system used to classify and scale injuries [22]. AIS codes are formed from six numbers that define the anatomical location of the injury, and the seventh number that defines the severity of injury. Full AIS code can also have two localizers, with two numbers each [22], but we did not use localizers in this analysis. Severity scale runs from 1 (minor) to 6 (maximal). Severity code 9 is used to describe unknown or unspecified severity. For example, injury code 751371.2 means an injury in body region upper extremity (7), anatomical structure of skeletal (5) and distal humerus fracture (13). Code 71 is not important in this case (code refers to complete articular; T-shaped; Y-shaped; T-condylar in that fracture) and the severity is 2 (moderate).

In this study injuries were aggregated to injury types used in the Burden of Disease studies (Table S1, Additional file 1) by developing an AIS-to-injury aggregation matrix. Each individual AIS code was assigned to an injury type with the guidance of the AIS Reporter [23] and expert judgment. The aggregation matrix from 1371 different AIS 2005 codes to injury types is in Additional file 2.

In cases where the exact injury type could not be defined, we excluded injuries with that AIS code from the calculation (76 815 injuries). The consequence of this exclusion was tested in a sensitivity analysis. Also all the injuries with victims over 100 years old were excluded (38 injuries) due to abnormally high age of these victims (150 years old, or older). After all the exclusions, the injury database had 181 757 injuries for 123 373 persons.

### Calculation of YLD

The YLDs for each victim was calculated by identifying the injury sustained and then multiplying the duration of that injury (in years) with the disability weight of that injury (between 0 and 1). For each victim we had information from the gender (i), age (j), mode of transport while injured (k), location (l) and year (m). See Table 1 for the description of the i, j, l and m, Table S2 (Additional file 1) for the description of k, and Table S1 (Additional file 1) for the description of duration and disability data.

Disability weight and duration data were obtained from the year 2008 update of the Burden of Disease study (Begg and Tomijima [18], based on Murray and Lopez [8]) (Table S1, Additional file 1). All injuries were assumed to be treated. In Begg and Tomijima [18] disability weights were estimated separately for five age categories and we used the average weight over these categories. The duration of lifelong injuries was based on the remaining life expectancy of the person. Thus, we assumed that a lifelong injury would not reduce the life-expectancy of the injured person. The remaining life expectancy data was based on the Coale and Demeny West level 26 life table, defined and used in the previous Burden of Disease studies [24]. We used same life table data in this study for comparison reason. In the West level 26 life table, the remaining life expectancy for males and females were 80.0 and 82.5 years, respectively, for the age group of 0 (Table S3, Additional file 1).

All YLDs were calculated separately for each individual victim. Only one injury per victim was included in the calculations to avoid unrealistically high YLDs for victims sustaining more than one lifelong injury. When victim had multiple injuries, we included the injury with highest YLDs and excluded the rest. Similar one injury per injured person approach was used e.g. in Dhondt et al. study [12].

The coverage of the STRADA data was taken into account when calculating total burden of injuries in Sweden by multiplying the YLDs with the reciprocal of the data coverage (Table 1). The population of Sweden for the study years is shown in Table S4 (Additional file 1).

### Calculation of YLL

STRADA had 1811 fatalities for the years 2007–2011. YLLs were calculated by comparing the age of persons who died in transport crashes to the expected life expectancy of that age and gender (Table S2, Additional file 1). For each victim we had information about the age (i), gender (j), mode of transport (k), location (l) and year (m). The coverage of fatality data was assumed to be 100% (Table 1).

## Results and discussion

### Burden due to injuries in Sweden, and the DALY rates

Injuries and fatalities due to transport crashes caused between 18 000 and 26 000 DALYs per year in Sweden in 2007–2011 (Table S4, Additional file 1). Approximately 41% of the DALYs were due to injuries (YLD) and the rest due to fatalities (YLL). The fraction of YLDs from DALYs varied between 33% and 49% between different years (Table S4, Additional file 1).

The YLDs due to injuries were mainly caused by lifelong injuries (Table S5, Additional file 1). Of all the injuries, only 2% caused lifelong health effects, but these lifelong injuries caused 96% of the total YLDs. For comparison, in Dhondt et al.’s [12] study in Belgium minor injuries contributed to 9% of YLDs.

Half of all DALYs in Sweden were sustained by people traveling by car (Table S6, Additional file 1). Pedestrians, cyclist and motorcyclists sustained 12%, 12% and 13%, respectively, of total DALYs. According to the national communication survey, in 2003–2004 the proportions of trips made by these three modes were 21%, 8% and 0.4%, respectively, and 60% of trips were made by car [25]. Other modes of transport had only minor contributions to the burden of injury.

The YLD rate was 96 per 100 000 inhabitants, with an annual variation between 77 and 117 per 100 000 inhabitant (Table S4, Additional file 1). The result is similar to the YLD rates for injuries estimated for the Rhône Département in France [16], Utrecht area in the Netherlands [15] and the Flanders and Brussels in Belgium [12]. In these three studies the YLDs per 100 000 inhabitants were 191, 120, 97, respectively. Beside the European Burden of injury study [17], which predicted YLD rates of 50 or less per 100 000 inhabitants for road injuries, the finding from previous studies support the results of our YLD calculation.

The average YLL rate was 142 per 100 000 inhabitants, with an annual variation between 104 and 192 per 100 000 inhabitant (Table S4, Additional file 1). The YLL rates per 100 000 inhabitants were 346, 270, 272 in Lapostolle et al., Holtslag et al. and Dhondt et al. [12, 15, 16], respectively, and between 200 and 350 YLLs per 100 000 inhabitants for males and between 50 and 120 YLLs per 100 000 inhabitants for females in the European burden of injury study [17]. According to European Union statistics, Sweden has the second lowest fatality rate per population due to road crashes, after the United Kingdom, which probably explains lower YLL rate in this study in comparison to previous studies [26].

### YLDs per injury

The average YLDs were 14.7, 0.012 and 0.27 for lifelong, temporal and all injuries, respectively, per crash per injured person (Table 2). From the different transport modes, the average YLDs for person for lifelong injuries was lowest for Pedestrians (9.4 YLDs) and highest for the mode of 'Other, unknown and missing’ (19.5 YLDs) (Table 2, see Additional file 1: Table S2, Additional material, for the definitions). Median YLDs were lowest for pedestrians and the highest for 'Other active travel modes’ (Table 2). Cyclist lost on average 31% less YLDs per lifelong injury than those injured in cars (both drivers and occupants) (Table 2). The result was statistically insignificant.

**Table 2:** **The average and median YLDs (SD = standard deviation) per injured person for different mode of transport, for lifelong, temporal and all injuries**

Mode	YLD per person (lifelong injuries)			YLD per person (temporal injuries)			YLD per person (all injuries)			Number of injured persons (lifelong injuries)	Number of injured persons (temporal injuries)
	Average	Median	SD	Average	Median	SD	Average	Median	SD	#	#
Pedestrian	9.4	6.3	8.0	0.0157	0.0188	0.0118	0.14	0.0188	1.4	500	38638
Bicycle	12.8	11.5	8.1	0.0131	0.0070	0.0115	0.24	0.0087	2.0	536	29289
Other active travel mode	14.7	20.0	8.6	0.0130	0.0087	0.0111	0.24	0.0160	2.1	28	1774
Moped	18.7	18.5	7.2	0.0105	0.0026	0.0111	0.43	0.0026	3.0	168	7383
Motorcycle	17.2	15.4	9.9	0.0146	0.0160	0.0119	0.69	0.0160	3.9	174	4251
Car	18.4	17.8	10.8	0.0071	0.0024	0.0099	0.34	0.0024	2.9	672	36451
Truck	15.3	13.4	8.0	0.0091	0.0026	0.0111	0.43	0.0026	2.8	27	949
Bus	10.2	6.5	9.0	0.0115	0.0026	0.0128	0.22	0.0026	1.9	23	1093
Other, unknown, missing	19.5	16.1	11.7	0.0134	0.0070	0.0125	0.55	0.0070	3.7	39	1378
**All modes**	**14.7**	**13.6**	**9.9**	**0.0120**	**0.0026**	**0.0117**	**0.27**	**0.0056**	**2.3**	**2167**	**121206**

Lifelong injuries in rural and urban area caused on average 17.0 and 13.0 YLDs per injured person, respectively (Table 3). From the different modes of transport, pedestrian injuries were the least serious in both areas. Cyclist sustained less severe injuries in both urban and rural areas when compared to those injured in car (both drivers and passengers) (Table 3).

**Table 3:** **The average and median YLDs (SD = standard deviation) per injured person for different mode of transport, injured in urban or rural location**

Mode	Urban			Rural		
	Average	Median	SD	Average	Median	SD
Pedestrian	9.4	6.2	8.2	9.5	6.9	7.2
Bicycle	12.4	11.2	7.8	14.3	12.9	9.2
Other active travel mode	14.4	16.1	8.7	20.9	20.9	1.3
Moped	19.0	18.5	7.3	17.5	18.2	6.5
Motorcycle	16.7	15.6	9.3	17.1	14.0	10.4
Car	20.1	20.2	11.1	18.1	17.3	11.0
Truck	16.3	20.3	9.5	14.9	13.3	7.6
Bus	9.7	6.0	9.3	12.2	15.5	8.0
Other, unknown, missing	20.2	19.3	12.3	18.9	15.7	11.6
**All modes**	**13.0**	**10.7**	**9.4**	**17.0**	**15.7**	**10.4**

Our findings for lifelong injuries are of the same magnitude as Dhondt et al. [12]. Dhondt et al. predicted median YLDs for drivers, passengers and slow modes (pedestrians, cyclist and mopeds) in Belgium. The resulting YLDs per road users were for lifelong injuries 10, 9.6, 7.1 and 8.7 YLDs per victim for drivers, passengers, slow mode, and all modes, respectively, when averaged over all the age groups. Our average YLDs for lifelong injuries are approximately twice as high for drivers and car occupants, and for all modes combined (Table 2). For slow modes the YLDs are similar when compared to average YLDs for pedestrians but smaller than YLDs for other active modes and mopeds. Our results for temporal injuries are approximately ten times smaller than YLDs in Dhondt et al. (Table 2). Dhondt et al. excluded slight injuries from their analysis which could explain the differences in results.

In another study done in the Netherlands the mean YLDs per injured person were 0.10 and 0.94 for patients treated in emergency department and for hospitalized patients, respectively [13]. The YLDs predicted in the Haagsma et al. [13] are in same magnitude with the average YLDs predicted for all the injuries combined (Table 2). Haagsma et al. [13] estimated YLDs for all non-intentional injuries and they used different injury types, duration and severity data than the present study.

**Figure 1:**
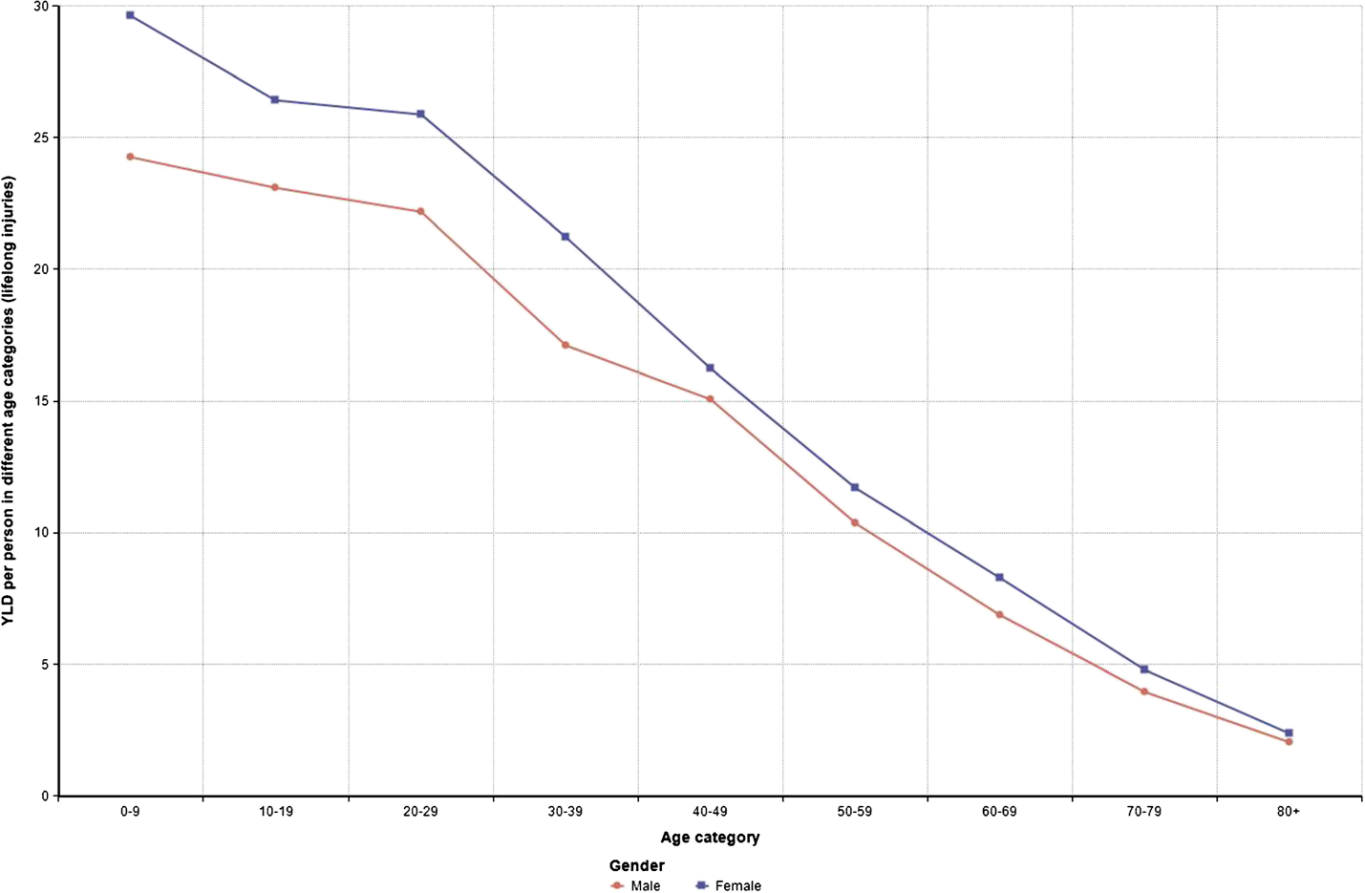
**YLDs per injured person in different age categories and gender (lifelong injuries).**

The average YLDs due to lifelong injuries were 14.6 for males and 15.0 for females per injured person (Table 4, see Table S7 in Additional file 1, for temporal injuries). For most transport modes females had higher average YLDs than males, with the exception of 'Other active travel’ and 'Bus’. The YLDs for different age categories followed similar pattern for both males and females so that average YLDs per injured person due to lifelong injuries were highest for the first age category (0–10 year old) and decreased in each following category (Figure 1). Females had higher average YLDs per injured person than males for all age groups.

**Table 4:** **The average and median YLDs (SD = standard deviation) per injured person for different mode of transport and gender (lifelong injuries)**

Mode	YLD per injured person (male)			YLD per injured person (female)		
	Average	Median	SD	Average	Median	SD
Pedestrian	9.1	6.3	7.8	9.6	6.2	8.3
Bicycle	12.3	10.5	7.9	13.8	12.3	8.4
Other active travel mode	16.6	21.1	8.7	8.9	8.6	5.6
Moped	17.7	17.5	7.1	21.9	23.8	6.5
Motorcycle	17.0	15.4	10.0	18.5	16.8	9.5
Car	17.4	17.5	9.8	20.4	19.2	12.3
Truck	14.1	13.2	6.3	22.3	21.7	13.6
Bus	11.9	8.9	10.5	8.7	5.7	7.5
Other, unknown, missing	17.6	15.4	10.9	26.7	25.5	12.3
**Average**	**14.6**	**13.9**	**9.4**	**15.0**	**12.6**	**10.9**

The risk taking behaviour of males has been studied in other fields of science [27, 28] and the consequences of this behaviour has been observed in previous burden of injury studies (e.g. Dhondt et al. [12], Lapostolle et al. [16], Polinder et al. [17]). In the present study we did not observe any clear gender difference in the average YLDs per injury caused by lifelong injuries. However, females had slighly higher average YLDs for lifelong injuries than males, for most modes (Table 4). The result could partly be explained by life expectancy variation between males and females (Table S3, Additional file 1).

Overall these results show that persons injured in different modes of transport have different kind of injuries, and these differences can potentially affect the burden of injury estimates for mode shifts. For example, a person who would shift in the urban area from car to bicycle would have on average 40% less severe injuries, if the person is injured to lifelong injury (Table 3). If the injury rate (ie the number of injuries per km driven and cycled) were the same for both modes, then the mode shift from car to bicycle would decrease the burden of lifelong injury by 40% (Table 3).

These changes in the severity of injuries are comparable to the injury rate differences. For example, in Pucher and Dijkstra [29] injury rates for cyclists were 1.3 (the Netherlands) to 12 (United States) times higher than injury rate for pedestrians, when the injury rate was expressed with injuries per km travelled. Thus, the 30% higher injury rate for cyclists, in comparison to pedestrians, in the Netherlands is of same magnitude as the severity difference of 40%, observed in this study.

### Severity classes

The YLDs for different severity classes is presented in Table 5. Minor crashes (with AIS-scale definition) contributed on average 0.01 YLDs per injury while maximal injuries caused over 2100 times more YLDs per injury. Serious injuries (AIS class 3) caused 58% of all the YLDs in Sweden.

**Table 5:** **The average and median YLDs (SD = standard deviation) per injury for different AIS severity classes**

	YLD per injury					
Severity	Average	Median	SD	Number of injuries	YLDs caused by injuries in Sweden in 2007-2011	%
**1. Minor**	0.012	0.003	0.305	71170	878	2.7%
**2. Moderate**	0.107	0.020	1.406	45620	4887	14.9%
**3. Serious**	3.322	0.052	7.362	5688	18896	57.8%
**4. Severe**	9.834	5.751	11.119	391	3845	11.8%
**5. Critical**	18.044	17.341	12.436	205	3699	11.3%
**6. Maximal**	25.363	25.363	16.935	2	51	0.2%
**Other**	1.519	0.009	6.867	297	451	1.4%
**Total**	-			-	32707	100%

The YLDs for severity classes of 4 and higher are of similar magnitude than the YLDs predicted in Holtslag et al.’s [15] study of injured trauma patients. In Holtslag et al. [15], the average YLD per major trauma patient was 12; major trauma was defined to be an injury with Injury Severity Score (ISS) higher than 15. ISS is the sum of the squares of MAIS (maximal AIS) values from the 3 most injured, arbitrary chosen body regions. For a person who suffers only one injury, an ISS over 15 corresponds to an injury that has an AIS severity score of 4, or more. In the present study the YLDs for AIS severity scores of 4, 5 and 6 were 9.8, 18.0 and 25.4, respectively (Table 5).

In the present study 3% of total YLDs were due to minor injuries (AIS = 1) (Table 5). McClure and Douglas’ [14] Australian study concluded that the AIS scale 1 injuries (minor injury) cause 80% of morbidity in the Australian Capital District area, when the health effects of injuries were summarized with the quality adjusted life-years (QALY) method. In McClure and Douglas [14], all injuries in the study area were collected by contacting medical doctors directly to capture all non-hospitalized injuries. It is likely that STRADA did not capture all the minor injuries and therefore the contribution of minor injuries is under predicted in the present study [30]. However, when the average YLD per injury for minor injuries is 0.01 YLDs, Sweden would need to have approximately 10 million minor injuries more (for the study period of five years) to increase the contribution of minor injuries to 80% from all the of YLDs; and much more if compared to DALYs.

It should also be noted that, when viewing the results for different severity classes, the severity codes were used to assess the injury types for different AIS codes, including the difference between temporal and lifelong injuries. Therefore the conclusions for different AIS codes should be viewed with certain caution due to possible circular reasoning.

### Contribution of different injury types

Approximately 60% of all YLDs due to transport crashes were due to intracranial injuries (Figure S1, Additional file 1). Injured spinal cords and fractures contributed 23% and 15%, respectively, of total YLDs.

When comparing different injury types between different modes of transport, some mode specific differences could be observed (Table S9, Additional file 1). For example, 25% of pedestrians had fractured radius or ulna while the motorized modes had 6%, or less of this injury type. Over half of the injuries were sprains for those injured in the cars.

In Lapostolle et al. [16] intracranial injuries caused 32% of YLDs, followed by injuries in spinal cord (32%) and fractures (31%). On the other hand, McClure and Douglas [14] concluded that half of the lifetime QALYs were due to sprains in age group of 16–34 year old, and in Polinder et al. [17] most important injury types were skull –brain and spinal cord injuries. Polinder et al. [17] included all injuries, so the numbers are only indicative for the transport injuries. Without details on the injury categorization and aggregation it is difficult to conclude how much the results of these different studies differ from each other.

A more detailed comparison of injury types between the present study, Lapostolle et al. [16] and Murray and Lopez [8] is shown in Table S10 (Additional file 1). Open wounds were the most common injuries in Lapostolle et al. [16] and after sprains second most common injury type in the present study. This is logical since almost all the AIS codes could be defined as open wounds. In Murray and Lopez [8] intracranial injuries were the most numerous injury types. Overall our study and the Lapostolle et al. [16] results are more similar than the injury distribution used in Murray and Lopez [8]. Figure S2 (Additional file 1) illustrates the consequence of these differences for the average YLD estimates of different studies. Based on Murray and Lopez [8], the average injury causes around 1 YLDs while the present and Lapostolle et al. [16] studies predict approximately one half to one fifth less YLDs per injury.

### Uncertainties and sensitivity of the results

Several assumptions were needed in different phases of the study to be able to calculate the results presented in previous chapters. Some of these assumptions are discussed in more details in the following paragraphs.

The calculation of YLDs was based on the aggregation of the AIS codes to injury categories. The AIS-to-injury aggregation matrix was created in this project following the guidance of the AIS Reporter [23] and expert judgment (see Additional file 2). This approach might over- or underestimates both the duration of the injury, and the type of the injury. To test the sensitivity of our results to AIS-to-injury aggregation, we made sensitivity analysis by assuming that some AIS-codes could be associated with other injury than what we used in main analyses (see Additional file 2). Table S11 (Additional file 1) shows the results of this sensitivity analyses for different modes for lifelong injuries. The difference between baseline analysis and sensitivity analysis results was less than 10% for most modes. This indicates that our results are robust for small AIS-to-injury aggregation uncertainty.

From the 1370 AIS codes used in the STRADA data, 142 were excluded from the calculations because we could not define exact injury type for those codes. As a result of this exclusion, 76 815 injuries (30%) were omitted from the analysis. From the 142 AIS codes omitted from the analysis, 16 had a severity weight of 9 (unknown or unspecified severity), 55 had a severity weight of 1 and 71 had a severity weight of over 1. As a sensitivity analysis we predicted the YLDs caused by all the injuries by assuming that each injury with AIS class of 1 would cause 0.01 YLDs, each injury with AIS class of 2 would cause 0.1 YLDs, and so on, based on the severity of the injury in AIS-scale (Table 5). When all injuries were included in the analysis, the average YLDs per injured person decreased by 12% and increased by 6% for males and females, respectively (Table S12, additional file 1). For pedestrians average YLDs increased by 72% and 128% for males and females, respectively, indicating that several undefined AIS-codes were related to injuries sustained by pedestrians. Similar but smaller changes in average YLDs were observed for all different modes of transport (Table S12, additional file 1).

In this study we used the same injury categories, severity weights and duration data as in the previous Burden of Disease studies [8, 18] and as in Lapostolle et al. [16]. Haagsma et al. [13] used different injury categories and analysed both duration and severity based on the data. They concluded that by using their newly defined injury categories, duration and severity data, the estimated burden of injury was 3 to 8 times higher than by using the standard method. However, in Haagsma et al. [13] the average YLDs per injury was similar than in the present study so it is unclear how the use of different injury types, duration and severity weights would have impacted our results.

The current study is based on the injury types, duration and severity data that has been used until recently in the burden of disease studies [8]. However, the recent burden of disease update published in the end of 2012 changed the injury types so that now all injuries, including traffic injuries, are divided to 23 injury sequelaes [31]. These sequelaes are estimated from the International Classification of Disease version 9 or 10 codes (ICD9 or ICD10) and by applying cause-nature matrix between injury types and the cause (such as road injury). Unfortunately, the methodological papers on the latest burden of disease approach [31, 32] did not include cause-nature matrixes for all the age groups, nor the duration of injury data. Without these data the comparison of methods cannot be done.

Our analysis was also solely based on the severity of injury without consideration of the exposure. In the case of transport injuries and fatalities, the exposure could be expressed e.g. with number of cases per distance travelled [33]. By combining the YLDs and YLLs predicted in this study with the exposure data we could estimate YLDs per distance travelled. One recent study from Belgium estimated DALYs per km driven for four different transport modes, showing that DALYs per km travelled vary greatly between transport modes [34]. However, the objective of this study was on the severity of injuries.

## Conclusions

We estimated the severity differences of injuries for different modes of transport and expressed these differences in YLDs. On average we observed two times difference in YLDs due to lifelong injuries depending on the transport mode that injured person was using while injured (Table 2). The injuries in rural areas were 1.3 times more severe than injuries in urban area (Table 3). We did not notice any significant gender differences in the average YLDs. The results of this study can be used to predict the health consequences of transport mode shifts in HIA and other similar studies.

## Electronic supplementary material

Additional file 1: **Additional tables and figures from the results.** PDF-file with twelve tables (S1-S12) and two figures (S1-S2) representing additional data and results. All the tables and figures are cited in the text. (PDF 158 KB)

Additional file 2: **AIS to Injury type conversation table.** Excel-file with the AIS to injury aggregation table. (XLSX 36 KB)

## Abbreviations

AIS: Abbreviated injury scale
DALY: Disability-adjusted life year
HIA: Health impact assessment
ICD: International classification of diseases
ISS: Injury severity score
MAIS: Maximal abbreviated injury scale
PM_2.5_: Fine particulate matter air pollution
QALY: Quality-adjusted life year
STRADA: Swedish traffic accident data acquisition
YLD: Years lost due to disability
YLL: Years of life lost.
