# Supplementary material for: Severity of injuries in different modes of transport, expressed with disability-adjusted life years (DALYs)
Source: BMC Public Health. 2014 Jul 29;14:765. doi: 10.1186/1471-2458-14-765 (PMC4131023; doi:10.1186/1471-2458-14-765)
Supplement: Supplementary file 1 — Additional file 1: Additional tables and figures from the results. PDF-file with twelve tables (S1-S12) and two figures (S1-S2) representing additional data and results. All the tables and figures are cited in the text. (PDF 158 KB) [file 12889_2014_6904_MOESM1_ESM.pdf]

Additional file 1 to Tainio et al. 2014 (BMC Public Health).

Table S1: Disability weights and duration of injury for different injury types (Begg and Tomijima 2006). For the injuries with duration of 'lifelong', the duration is same as the expected life expectancy of the person with same age and gender (see Table S3, Additional file 2).

Injury type	Disability weight	Duration (year)
Fractured skull	0.431	0.107
Fractured skull (lifelong)	0.361	Lifelong
Fractured face bones	0.223	0.118
Fractured vertebral column	0.226	0.14
Fractured rib or sternum	0.199	0.115
Fractured pelvis	0.247	0.126
Fractured clavicle, scapula or humerus	0.143	0.112
Fractured radius or ulna	0.18	0.112
Fractured hand bones	0.1	0.07
Fractured femur	0.372	0.14
Fractured femur (lifelong)	0.272	Lifelong
Fractured patella, tibia or fibula	0.271	0.09
Fractured ankle	0.196	0.096
Fractured foot bones	0.077	0.073
Injured spinal cord (lifelong)	0.725	Lifelong
Dislocations shoulder, elbow or hip	0.074	0.034
Dislocations other dislocation	0.074	0.019
Sprains	0.064	0.038
Intracranial injuries	0.359	0.067
Intracranial injuries (lifelong)	0.361	Lifelong
Internal injuries	0.208	0.042
Open wound	0.108	0.024
Injury to eyes	0.108	0.019
Injury to eyes (lifelong)	0.299	Lifelong
Amputations, thumb, finger (lifelong)	0.134	Lifelong
Amputations, arm (lifelong)	0.257	Lifelong
Amputations, toe (lifelong)	0.102	Lifelong
Amputations, foot, leg (lifelong)	0.3	Lifelong
Crushing	0.218	0.094
Burns <20%	0.158	0.083
Burns >20% (lifelong)	0.255	Lifelong
Burns <20% (lifelong)	0.001	Lifelong
Burns >20%	0.441	0.279
Injured nerves (lifelong)	0.064	Lifelong
Poisoning	0.909	0.008

Table S2: Description of the transport modes used in the study. Number in the parenthesis refers to the STRADA code of the mode.

**Mode of transport**	**Sub-modes**
Pedestrian	Pedestrian (10)
Bicycle	Bicycle (20)
Other active travel mode	Roller skates (11), wheel chair (12), skateboard (13), kick bike (14, 15) and other pedestrian (16)
Moped	Moped (30) and scooter (31)
Motorcycle	Motorcycle (40), heavy motorcycle (41) and light motorcycle (42)
Car	Passenger car (50) and minivan (51)
Truck	Truck (60), heavy truck (61) and light truck (62)
Bus	Bus (70)
Other, unknown, missing	Other (80), snowmobile (81), tram (82), tractor (83), train (84), rider (85), all-terrain vehicle (86), unknown (90), missing (0)

Table S3: Remaining life expectancy at different age and gender. The life expectations are based on a model life table, called Coale and Demeny West level 26 (Murray 1994). Life expectancy data was used to calculate the loss of life expectancy (YLL) and the duration of injuries for lifelong injuries.

	Life expectancy			Life expectancy	
**Age**	**Male**	**Female**	**Age**	**Male**	**Female**
**0**	80.00	82.50	**51**	30.06	33.07
**1**	79.36	81.84	**52**	29.12	32.14
**2**	78.36	80.87	**53**	28.19	31.22
**3**	77.37	79.90	**54**	27.26	30.29
**4**	76.38	78.92	**55**	26.32	29.37
**5**	75.38	77.95	**56**	25.42	28.46
**6**	74.39	76.96	**57**	24.52	27.55
**7**	73.39	75.97	**58**	23.61	26.65
**8**	72.39	74.97	**59**	22.71	25.74
**9**	71.40	73.98	**60**	21.81	24.83
**10**	70.40	72.99	**61**	20.95	23.95
**11**	69.40	72.00	**62**	20.09	23.07
**12**	68.41	71.00	**63**	19.22	22.20
**13**	67.41	70.01	**64**	18.36	21.32
**14**	66.41	69.01	**65**	17.50	20.44
**15**	65.41	68.02	**66**	16.71	19.59
**16**	64.42	67.03	**67**	15.93	18.74
**17**	63.42	66.04	**68**	15.15	17.90
**18**	62.43	65.06	**69**	14.36	17.05
**19**	61.43	64.07	**70**	13.58	16.20
**20**	60.44	63.08	**71**	12.89	15.42
**21**	59.44	62.10	**72**	12.21	14.63
**22**	58.45	61.12	**73**	11.53	13.85
**23**	57.46	60.13	**74**	10.85	13.06
**24**	56.46	59.15	**75**	10.17	12.28
**25**	55.47	58.17	**76**	9.62	11.60
**26**	54.48	57.19	**77**	9.08	10.93
**27**	53.49	56.21	**78**	8.53	10.25
**28**	52.50	55.23	**79**	7.99	9.58
**29**	51.50	54.25	**80**	7.45	8.90
**30**	50.51	53.27	**81**	7.01	8.36
**31**	49.52	52.29	**82**	6.56	7.83
**32**	48.53	51.31	**83**	6.12	7.29
**33**	47.54	50.34	**84**	5.68	6.76
**34**	46.55	49.36	**85**	5.24	6.22
**35**	45.57	48.38	**86**	4.90	5.83

**36**	44.58	47.41	**87**	4.56	5.43
**37**	43.60	46.44	**88**	4.22	5.04
**38**	42.61	45.47	**89**	3.88	4.64
**39**	41.63	44.50	**90**	3.54	4.25
**40**	40.64	43.53	**91**	3.30	3.98
**41**	39.67	42.57	**92**	3.05	3.71
**42**	38.69	41.61	**93**	2.80	3.43
**43**	37.72	40.64	**94**	2.56	3.16
**44**	36.74	39.68	**95**	2.31	2.89
**45**	35.77	38.72	**96**	2.14	2.71
**46**	34.81	37.77	**97**	1.97	2.53
**47**	33.86	36.83	**98**	1.80	2.36
**48**	32.90	35.88	**99**	1.63	2.18
**49**	31.95	34.94	**100**	1.46	2.00
**50**	30.99	33.99			

Table S4: DALYs, YLDs and YLLs due to transport injuries and fatalities in Sweden in 2007-2011. The YLDs in the 'YLD (Sweden)' row are adjusted for the data coverage. See text for details.

**Year**	**2007**	**2008**	**2009**	**2010**	**2011**	**Average**
**Population of Sweden**	9,147,057	9,216,748	9,256,347	9,340,682	9,415,570	9,275,281
**YLD (based on data)**	5,589	7,513	6,073	7,756	6,422	-
**Coverage of population in injury data**	64%	70%	74%	82%	89%	-
**YLD (Sweden)**	8,713	10,762	8,193	9,485	7,251	8,881
**YLL (Sweden)**	17,531	14,273	13,030	9,748	11,182	13,153
**DALY (Sweden) (YLD+YLL)**	26,244	25,036	21,223	19,233	18,433	22,034
**Percentage of DALYs due to YLDs**	33%	43%	39%	49%	39%	41%
**YLDs per 100 000 inhabitants**	95	117	89	102	77	96
**YLLs per 100 000 inhabitants**	192	155	141	104	119	142

Table S5: Contribution of lifelong and temporal injuries to total YLDs in Sweden.

	Number of injuries		YLDs	
**Injury type**	**#**	**%**	**YLDs**	**%**
Lifelong injury	2,167	2%	31,900	96%
Temporal injury	121,206	98%	1,454	4%
**All injuries**	**123,373**	**100%**	**33,354**	**100%**

Table S6: Average DALYs, YLDs and YLLs in Sweden due to injuries (YLDs) and fatalities (YLLs) sustained in different mode of transport. The results are averaged over the five study years. YLDs presented in this table are adjusted for the data coverage. See text for details.

**Mode of transport**	**YLD (injury)**	**YLL (deaths)**	**DALY (YLD+YLL)**	**% (DALY)**
**Pedestrian**	1,391	1,237	2,628	12%
**Bicycle**	1,933	636	2,569	12%
**Other active travel mode**	109	0	109	0%
**Moped**	870	505	1,375	6%
**Motorcycle**	816	2,038	2,855	13%
**Car**	3,378	7,811	11,189	51%
**Truck**	113	537	650	3%
**Bus**	66	75	141	1%
**Other, unknown, missing**	205	313	518	2%
**Total**	**8,881**	**13,153**	**22,034**	**100%**

Table S7: The average and median YLDs (SD = standard deviation) per injured person for different mode of transport and gender (temporal injuries).

**Mode of transport**	**YLD per injured person (male)**			**YLD per injured person (female)**		
	Average	Median	SD	Average	Median	SD
Pedestrian	0.0143	0.0160	0.0127	0.0164	0.0202	0.0114
Bicycle	0.0129	0.0070	0.0115	0.0134	0.0160	0.0116
Other active travel mode	0.0123	0.0070	0.0107	0.0144	0.0160	0.0116
Moped	0.0115	0.0056	0.0115	0.0087	0.0026	0.0100
Motorcycle	0.0148	0.0160	0.0120	0.0132	0.0070	0.0111
Car	0.0077	0.0024	0.0103	0.0065	0.0024	0.0093
Truck	0.0092	0.0026	0.0112	0.0079	0.0024	0.0098
Bus	0.0096	0.0026	0.0116	0.0124	0.0026	0.0133
Other, unknown, missing	0.0133	0.0070	0.0127	0.0135	0.0070	0.0122
**Average**	**0.0114**	**0.0026**	**0.0118**	**0.0126**	**0.0070**	**0.0116**

Table S8: The average and median YLDs (SD = standard deviation) per injured person for different age categories and gender (lifelong injuries). For graphical presentation of averages YLDs, see Figure 1.

	YLD per injured person (male)			YLD per injured person (female)				
Age category	Average	Median	SD	Average	Median	SD	Number of lifelong injuries (male)	Number of lifelong injuries (female)
0-9	24.3	27.2	7.3	29.6	28.1	7.3	12	14
10-19	23.1	22.9	6.9	26.4	24.2	8.3	227	115
20-29	22.2	20.4	8.7	25.9	22.1	9.5	258	102
30-39	17.1	16.1	6.7	21.2	18.2	8.0	178	71
40-49	15.1	13.3	5.6	16.3	14.3	6.1	161	70
50-59	10.4	9.5	3.2	11.7	10.6	4.3	175	93
60-69	6.9	6.6	2.2	8.3	7.7	2.7	171	114
70-79	4.0	3.9	1.0	4.8	4.7	1.4	122	111
80+	2.1	2.1	0.5	2.4	2.4	0.7	97	76

Table S9: Percentage of different injuries in different modes of transport.

Injury type	Pedestrian	Bicycle	Other active travel mode	Moped	Motor-cycle	Car	Truck	Bus	Other, unknown, missing
Fractured skull	0.2%	0.5%	0.7%	0.3%	0.5%	0.2%	0.5%	0.3%	0.6%
Fractured skull (lifelong)	0.1%	0.1%	0.2%	0.1%	0.2%	0.1%	0.2%	0.1%	0.0%
Fractured face bones	2.3%	5.3%	2.7%	1.9%	1.6%	1.4%	1.8%	1.3%	2.0%
Fractured vertebral column	0.8%	1.1%	0.4%	1.0%	5.4%	3.7%	6.3%	4.5%	4.4%
Fractured rib or sternum	1.0%	1.9%	0.7%	1.6%	7.4%	3.2%	3.5%	3.8%	3.5%
Fractured pelvis	1.3%	1.0%	0.5%	0.6%	1.7%	0.6%	1.1%	1.3%	1.7%
Fractured clavicle, scapula or humerus	8.4%	7.9%	7.5%	6.4%	10.0%	0.9%	2.0%	3.8%	5.9%
Fractured radius or ulna	24.9%	13.2%	23.2%	6.0%	6.1%	0.8%	1.4%	4.4%	8.1%
Fractured hand bones	4.9%	6.6%	8.7%	4.8%	6.6%	0.9%	1.1%	2.6%	4.5%
Fractured femur	4.1%	2.1%	2.1%	1.7%	2.1%	0.5%	0.6%	3.3%	2.8%
Fractured femur (lifelong)	0.1%	0.1%	0.0%	0.6%	1.2%	0.2%	0.3%	0.1%	0.6%
Fractured patella, tibia or fibula	7.0%	3.3%	3.4%	4.9%	7.7%	0.7%	1.1%	2.1%	3.5%
Fractured ankle	5.0%	1.4%	1.8%	1.7%	2.5%	0.2%	0.2%	0.7%	2.0%
Fractured foot bones	1.6%	1.1%	1.7%	2.7%	3.0%	0.3%	1.0%	1.3%	0.7%
Injured spinal cord (lifelong)	0.1%	0.1%	0.0%	0.1%	0.7%	0.4%	0.3%	0.4%	0.6%
Dislocations shoulder, elbow or hip	1.3%	1.5%	1.5%	0.6%	1.7%	0.1%	0.4%	0.4%	1.3%
Dislocations other	1.0%	0.7%	0.9%	0.4%	0.3%	0.3%	0.2%	0.6%	0.4%

dislocation									
Sprains	15.0%	10.3%	14.3%	10.8%	12.9%	60.0%	41.5%	33.5%	22.7%
Intracranial injuries	6.5%	10.1%	6.1%	9.0%	6.3%	6.6%	7.2%	10.6%	10.8%
Intracranial injuries (lifelong)	1.1%	1.5%	1.4%	1.4%	1.8%	1.1%	1.9%	1.3%	1.6%
Internal injuries	0.1%	0.6%	0.6%	0.9%	1.5%	0.9%	1.5%	0.4%	1.1%
Open wound	13.2%	29.6%	21.5%	42.2%	18.6%	16.6%	25.4%	23.1%	21.1%
Injury to eyes	0.0%	0.0%	0.1%	0.0%	0.0%	0.1%	0.0%	0.1%	0.1%
Injury to eyes (lifelong)	0.0%	0.0%	0.0%	0.0%	0.0%	0.1%	0.1%	0.0%	0.0%
Amputations, thumb, finger (lifelong)	0.0%	0.0%	0.1%	0.0%	0.1%	0.0%	0.0%	0.0%	0.0%
Amputations, arm (lifelong)	0.0%	0.0%	0.0%	0.0%	0.0%	0.0%	0.0%	0.0%	0.0%
Amputations, toe (lifelong)	0.0%	0.0%	0.0%	0.0%	0.0%	0.0%	0.0%	0.0%	0.0%
Amputations, foot, leg (lifelong)	0.0%	0.0%	0.0%	0.0%	0.0%	0.0%	0.0%	0.1%	0.0%
Crushing	0.0%	0.0%	0.1%	0.0%	0.0%	0.0%	0.0%	0.0%	0.1%
Burns <20%	0.0%	0.0%	0.0%	0.1%	0.1%	0.1%	0.0%	0.0%	0.1%
Burns >20% (lifelong)	0.0%	0.0%	0.0%	0.0%	0.0%	0.0%	0.1%	0.1%	0.0%
Burns <20% (lifelong)	0.0%	0.0%	0.0%	0.0%	0.0%	0.0%	0.0%	0.0%	0.0%
Burns >20%	0.0%	0.0%	0.0%	0.0%	0.0%	0.0%	0.0%	0.0%	0.0%
Injured nerves (lifelong)	0.0%	0.0%	0.0%	0.0%	0.1%	0.0%	0.0%	0.1%	0.0%
Poisoning	0.0%	0.0%	0.0%	0.0%	0.0%	0.0%	0.0%	0.0%	0.0%
Sum	100.0%	100.0%	100.0%	100.0%	100.0%	100.0%	100.0%	100.0%	100.0%

Table S10: Percentage of different injury types in the present, Lapostolle et al. (2009) and Murray and Lopez (1996) studies.

	Present (male)	Present (female)	Lapostolle et al. 2009 (male)	Lapostolle et al. 2009 (female)	Murray and Lopez 1996
Fractured skull	0.4%	0.2%	0.5%	0.3%	1.8%
Fractured skull (lifelong)	0.1%	0.1%	0.1%	0.1%	0.3%
Fractured face bones	2.9%	2.5%	2.2%	1.7%	3.1%
Fractured vertebral column	2.6%	1.4%	1.4%	1.4%	3.1%
Fractured rib or sternum	3.0%	1.4%	3.0%	4.0%	3.0%
Fractured pelvis	0.8%	1.1%	1.1%	1.5%	2.2%
Fractured clavicle, scapula or humerus	5.7%	6.0%	4.4%	2.8%	5.1%
Fractured radius or ulna	7.3%	17.4%	4.8%	4.1%	5.8%
Fractured hand bones	4.8%	3.5%	3.4%	1.6%	1.1%
Fractured femur	2.1%	2.4%	1.0%	0.5%	7.3%
Fractured femur (lifelong)	0.3%	0.1%	0.7%	0.3%	0.4%
Fractured patella, tibia or fibula	3.5%	4.3%	3.4%	2.3%	9.4%
Fractured ankle	1.8%	2.7%	1.6%	1.2%	3.2%
Fractured foot bones	1.1%	1.3%	1.7%	1.1%	0.9%
Injured spinal cord (lifelong)	0.2%	0.2%	0.4%	0.5%	1.5%
Dislocations shoulder, elbow or hip	1.3%	0.7%	1.5%	0.7%	1.5%
Dislocations other dislocation	0.6%	0.6%	16.6%	37.2%	0.0%
Sprains	26.2%	28.9%	7.4%	5.6%	2.5%
Intracranial injuries	8.0%	7.2%	0.8%	0.5%	35.7%
Intracranial injuries (lifelong)	1.6%	0.9%	0.9%	0.5%	1.9%
Internal injuries	0.8%	0.4%	2.9%	1.8%	2.7%
Open wound	24.4%	16.7%	39.5%	30.0%	5.4%
Injury to eyes	0.1%	0.0%	0.3%	0.3%	0.0%
Injury to eyes (lifelong)	0.0%	0.0%	0.0%	0.0%	0.1%
Amputations, thumb, finger (lifelong)	0.0%	0.0%	0.0%	0.0%	0.1%
Amputations, arm (lifelong)	0.0%	0.0%	0.0%	0.0%	0.0%
Amputations, toe (lifelong)	0.0%	0.0%	0.0%	0.0%	0.0%

Amputations, foot, leg (lifelong)	0.0%	0.0%	0.0%	0.0%	0.1%
Crushing	0.0%	0.0%	0.1%	0.1%	0.8%
Burns <20%	0.1%	0.0%	0.0%	0.0%	0.0%
Burns >20% (lifelong)	0.0%	0.0%	0.0%	0.0%	0.0%
Burns <20% (lifelong)	0.0%	0.0%	0.0%	0.0%	0.1%
Burns >20%	0.0%	0.0%	0.0%	0.0%	0.0%
Injured nerves (lifelong)	0.0%	0.0%	0.1%	0.0%	0.2%
Poisoning	0.0%	0.0%	0.0%	0.0%	0.6%
Total	100.0%	100.0%	100.0%	100.0%	100.0%

Table S11: Sensitivity analysis. YLDs per person with alternative AIS-codes presented in Additional file 1, and the comparison to baseline results. See text for details.

	**Average YLDs per person, alternative AIS-to-injury matrix (lifelong injuries)**		**Average YLDs per person, main results (lifelong injuries)**		**Difference**	
**Mode of transport**	**Average**	**Median**	**Average**	**Median**	**Average**	**Median**
Pedestrian	8.8	6.2	9.4	6.3	94%	97%
Bicycle	12.5	11.3	12.8	11.5	98%	98%
Other active travel mode	14.4	19.9	14.7	20.0	98%	100%
Moped	18.9	22.1	18.7	18.5	101%	119%
Motorcycle	15.6	15.4	17.2	15.4	91%	100%
Car	17.6	18.1	18.4	17.8	95%	102%
Truck	14.7	12.6	15.3	13.4	96%	94%
Bus	9.6	6.7	10.2	6.5	94%	104%
Other, unknown, missing	16.8	15.7	19.5	16.1	86%	98%

Table S12: Sensitivity analysis. YLDs per person with and without 76 815 injuries for which the exact injury type could not be defined. See text for details.

	**Average YLDs per person, main results**		**Average YLD per person, all injuries**		**Difference**	
**Mode of transport**	**Male**	**Female**	**Male**	**Female**	**Male**	**Female**
Pedestrian	0.19	0.11	0.33	0.25	172%	228%
Bicycle	0.27	0.21	0.27	0.20	100%	93%
Other active travel mode	0.31	0.11	0.23	0.22	74%	194%
Moped	0.46	0.37	0.34	0.14	75%	40%
Motorcycle	0.70	0.63	0.70	0.45	101%	70%
Car	0.39	0.28	0.23	0.16	60%	58%
Truck	0.38	0.93	0.32	0.36	84%	38%
Bus	0.38	0.15	0.18	0.27	48%	177%
Other, unknown, missing	0.56	0.52	0.50	0.36	89%	70%
**Average**	**0.34**	**0.20**	**0.30**	**0.21**	**88%**	**106%**

Figure S1: The fraction of YLDs due to different injury types.

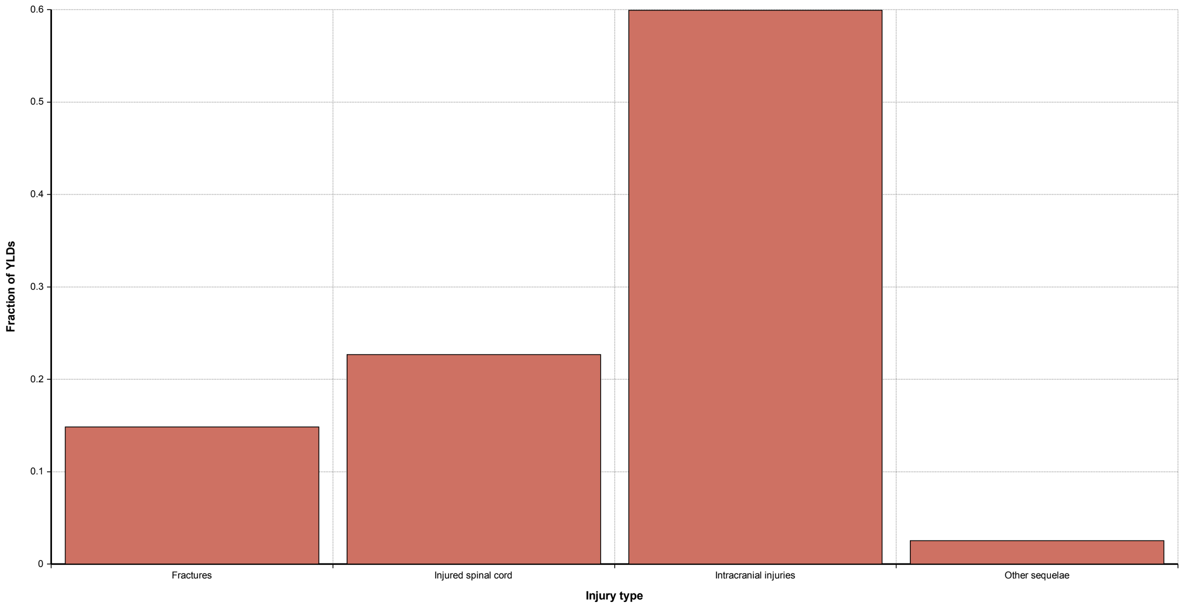

Figure S2: YLD per injury in different studies.

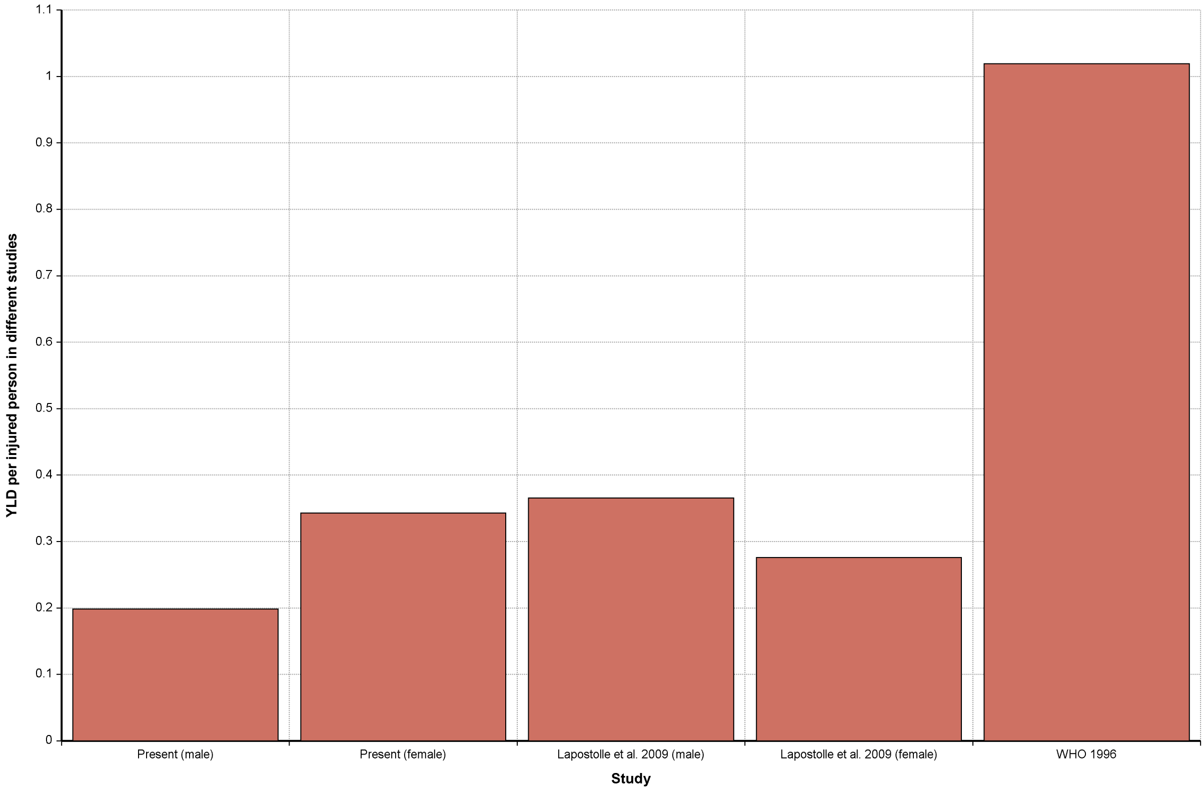

References

Begg S, Tomijima N: Global burden of injury in the year 2000: an overview of methods. World Health Organization (WHO); 2006:1–22.

Lapostolle A, Gadegbeku B, Ndiaye A, Amoros E, Chiron M, Spira A, Laumon B: The burden of road traffic accidents in a French Departement: the description of the injuries and recent changes. BMC Public Health 2009, 9.

Murray CJL, Lopez AD: The global burden of disease: a comprehensive assessment of mortality and disability from diseases, injuries, and risk factors in 1990 and projected to 2020. Cambridge, MA: Published by the Harvard School of Public Health on behalf of the World Health Organization and the World Bank ; Distributed by Harvard University Press; 1996.

Murray CJL: Quantifying the Burden of Disease – The technical basis for disability-adjusted life years. Bull. World Health Organ. 1994, 72:429–445.
